# Anxiety and prior victimization predict online gender-based violence perpetration among Indonesian young adults during COVID-19 pandemic: cross-sectional study

**DOI:** 10.1186/s41935-022-00292-4

**Published:** 2022-07-07

**Authors:** Gede Benny Setia Wirawan, Magdalena Anastasia Hanipraja, Gabrielle Chrysanta, Nadya Imtaza, Karima Taushia Ahmad, Inda Marlina, Dimas Mahendra, Alvin Theodorus Larosa

**Affiliations:** 1 Tabu Indonesia Berdaya Foundation, Jakarta, Indonesia; 2 Center for Public Health Innovation, Faculty of Medicine, Udayana University, Denpasar, Indonesia; 3 Hematology Department, University of Alberta Hospital, Edmonton, Canada; 4 Faculty of Public Health, University of Indonesia, Jakarta, Indonesia; 5 Faculty of Social and Political Sciences, University of Indonesia, Jakarta, Indonesia

**Keywords:** Gender-based violence, Cyber violence, Perpetration, Young adult, Indonesia

## Abstract

**Background:**

Most of human interactions moved to the cyberspace for much of the pandemic. It was no surprise that online violence was also on the rise. One of the objectives of this study was to describe the prevalence and risk factors of online gender-based violence (OGBV) perpetration during the COVID-19 pandemic.

**Results:**

The final analysis included 1006 respondents, 84.2% of whom were women and 94.5% were heterosexual. Over 60% of respondents admitted having perpetrated at least one type of OGBV once. It included 58.6% of women who admitted having perpetrated OGBV. Logistic regression analysis identified anxiety, online disinhibition, and history of victimization as independent risk factors of perpetration with an adjusted odds ratio (aOR) of 1.82 (95% CI 1.30–2.56), 1.38 (95% CI 1.03–1.85), and 9.72 (95% CI 5.11–18.51), respectively. Sub-group analysis that identified these factors also facilitated increased frequency and severity of OGBV perpetration.

**Conclusions:**

We found a high proportion of OGBV perpetration among young adults during the pandemic among all genders although women were grossly overrepresented among the respondents. Risk factors of perpetration included anxiety, online disinhibition, and prior victimization. The pandemic situation which heightened general anxiety and increased dependency on online communication may facilitate the perpetration of OGBV. The generalization of this result should pay attention to the caveat that the demographic of respondents is heavily skewed toward women.

## Background

Online gender-based violence (OGBV) is an emerging concern among academics and feminist activists. Its theoretical framework stems from gender-based violence (GBV), which was defined by the United Nations as acts of violence motivated by the gender of the victims, likely to result in physical, sexual, or psychological harm or suffering (United Nations 1993). As the definition suggests, GBV is not limited to physical violence. Indeed, the Internet has been proven to be the perfect medium for it (Suzor et al. 2019). One study has attempted to operationalize OGBV based on the most frequently reported acts which include unwanted messages, harassment, exposure to unwanted explicit materials, doxxing, and even sexual threats or exploitation (Vitak et al. 2017; Dunn 2020).

As more and more of our activities are being conducted online, the incidence of OGBV has become more prevalent. Like GBV, women are the primary victims of OGBV. This is especially true for prominent women and public figures. Two studies have described the vulnerability of female academics and athletes to online hate (Kavanagh et al. 2019; Kavanagh and Brown 2020). These women faced different forms of OGBV, which highlight the diverse forms of OGBV. Online hate targeting female academics was mostly gatekeeping: keeping women out of mostly male-dominated fields (Kavanagh and Brown 2020). Meanwhile, athletes mostly faced explicit, vile, and unwanted sexualization (Kavanagh et al. 2019). Despite these examples, OGBV is by no means limited to public figures or otherwise prominent women. A survey found that 20–40% of female university students in the USA have experienced some form of OGBV at least once (Vitak et al. 2017).

The COVID-19 pandemic has only worsened the GBV situation. A scoping review in the first few months of the pandemic highlighted an increased report of GBV cases, especially in domestic settings (Dlamini 2021). This is also true for OGBV. A report from Indonesian National Commission on Violence Against Women found a 300% increase in the number of cases in the pandemic year 2020 (National Commission Against Violence on Women 2020). A similar increase has also been described in other countries which indicates that this is a global phenomenon (UN Women 2020). This issue is especially relevant to young people who dominate Internet usage in Indonesia as well as globally (Indonesian Internet Service Provider Association 2020).

There have been some studies conducted on the risk factors and impacts of OGBV victimizations. Prominence in public spaces has been described as a risk factor for OGBV victimization (Kavanagh et al. 2019; Kavanagh and Brown 2020). This is especially true for female rights activists (Jatmiko and Syukron 2020). Studies have found that OGBV victimizations exert negative psychological (e.g., depression, social anxiety), social (e.g., social isolation), and reproductive consequences on the victims (UN Women 2020) (Cripps and Stermac 2018).

Previous studies have identified the risk factors of GBV and online abuse perpetration (Philpart et al. 2009; Kang et al. 2021). Despite these discoveries, few studies have described the risk factors of OGBV perpetration as a separate phenomenon. Designing effective interventions against OGBV requires an understanding of the factors that facilitates the risk of perpetrating OGBV (Decker et al. 2013). As such, the objectives of our study are to fill in this gap of knowledge and to elucidate the risk factors of OGBV perpetration.

## Methods

### Study settings

Data collection was conducted through social media platforms between July and August 2021, approximately 16 months after the first identified COVID-19 case in Indonesia. While the central government has relaxed its enforcement of the lockdown policy, lockdown at the municipal level was still implemented. Most employers and educational institutions still conduct activities mostly or entirely online. Accordingly, Indonesia experienced an explosion in Internet usage. Internet users in Indonesia increased by 8.6% in the second quarter of 2020 and increased further by 15.5% in the second quarter of 2021, just prior to data collection (Riyanto 2021).

Regarding OGBV, the Indonesian National Commission on Violence Against Women released its annual report a few months prior to data collection. The report highlighted an increased number of GBV cases, including OGBV, during the pandemic (National Commission Against Violence on Women 2020). The story was taken up by mainstream media (Pratiwi 2021). Several community organizations also organized social media awareness campaigns (Ratnasari et al. 2021). As such, awareness of OGBV may be already increased at the time of data collection.

### Study design and data collection

This study employed a cross-sectional analytic design. Data was collected using an online survey built in the Indonesian language on the Google Form platform. The survey was disseminated through our institution’s social media channels with assistance from collaborating civil organizations (e.g., student councils at several universities in Indonesia). The survey was open for 4 weeks. Ten respondents were randomly selected to receive a financial incentive of IDR 50,000 per person (equal to USD 3). The inclusion criterion for the analysis was young adults (aged 18 to 35 years old) who have lived in Indonesia for at least a year prior to the data collection period. Respondents were dropped from the analysis if they were unwilling to submit the last 6 digits of their phone number as identifiers in the analysis.

### Variables and measures

One of the variables of interest in our study is OGBV perpetration, including the type and frequency of perpetration. This variable was measured using a modified version of the OGBV victimization frequency measurement instrument devised by Vitak et al. (2017). The instrument included several forms of OGBV from incessant messaging, threats and insults, and unwanted pornographic messages to doxxing. The frequency of perpetration was measured by a 5-point Likert scale ranging from 0 (“never”) to 4 (“very often”). In recognition of the different degrees of impacts, each of these actions may cause to the victim, scoring for each item was weighted accordingly. The weight applied to each item refers to the weights identified in a study conducted by Vitak et al. (2017). Items that represent more serious actions received larger weights compared to those that represent less serious actions. Thus, the final OGBV perpetration score indicates both the severity and frequency of OGBV actions perpetrated.

Variables proposed as risk factors of OGBV perpetration in this study were psychological factors: mental distress, intolerance of uncertainty, and online disinhibition. Mental distress was measured using the Depression, Anxiety, and Stress Scale (DASS-21). Each subscale was scored separately and categorized based on a cutoff point described by Lovibond and Lovibond (Lovibond and Lovibond 1995). The DASS-21 questionnaire has been translated into the Indonesian language by Damanik. The translated instrument has undergone reliability and validity analysis (Damanik 2011).

Intolerance of uncertainty was measured using the 12-item short version of the Intolerance of Uncertainty Scale (IUS) devised by Carleton et al. (2007). Online disinhibition was measured by an Online Disinhibition Scale (ODS) adapted by Febriana and Febriana and Fajrianthi (2019). Both measures were translated and back-translated by our research team. The reliability of the translated measures was analyzed with Cronbach’s alpha value for IUS and ODS respectively of 0.854 and 0.889.

We also measured covariates that included the history of OGBV victimization and demographic characteristics, including gender, age, and sexual orientation. OGBV victimization history was measured as binary (yes or no) based on the items from the aforementioned OGBV perpetration measurement (Vitak et al. 2017). Respondents were classified as having a history of OGBV victimization if they answered yes to any of the items.

### Data analysis

The primary analysis was conducted using univariate and multivariate binomial logistic regressions to identify how the risk factors proposed are linked with the odds of ever committing OGBV. The effect size for logistic regression was presented as crude odds ratio (cOR) and adjusted odds ratio (aOR) for univariate and multivariate analysis, respectively. Further sub-group analysis was conducted for respondents who admitted OGBV perpetration. The sub-group analysis was conducted using multivariate linear regression. It aims to understand the factors associated with higher/lower frequency and severity of the OGBV committed. All analyses were conducted using IBM SPSS 23.0 (Armonk, NY, USA) for Microsoft Windows.

## Results

Data collection initially received 1079 responses. After the data cleaning process, the final analysis included 1006 respondents (Table 1). Most respondents identified as women (84.2%), with only 15.5% identified as men and 0.3% being non-binary. Nearly all respondents (94.5%) identified as heterosexual. Meanwhile, the median age of the respondents was 26 years old, and the interquartile range is 22 to 30 years old. The respondents’ ages were not normally distributed.

**Table 1:** Characteristics of respondents

Variables (*n* = 1006)	
Age (years), median (IQR)	26 (22–30)
Gender, *n* (%)	
Men	156 (15.5)
Women	847 (84.2)
Non-binary	3 (0.3)
Sexual orientation, *n* (%)	
Heterosexual	955 (94.5)
Homosexual	6 (0.6)
Bisexual	7 (0.7)
Asexual	17 (1.7)
Questioning	21 (2.1)
Depression category, *n* (%)	
No depression	812 (80.7)
Mild	123 (12.2)
Moderate	69 (6.9)
Severe	2 (0.2)
Extreme	0 (0.0)
Anxiety category, *n* (%)	
Non-anxiety	589 (58.5)
Mild	155 (15.4)
Moderate	203 (20.2)
Severe	58 (5.8)
Extreme	1 (0.1)
Stress category, *n* (%)	
No stress	914 (90.9)
Mild	74 (7.4)
Moderate	18 (1.8)
Severe	0 (0.0)
Extreme	0 (0.0)
Intolerance of uncertainty, *n* (%)	
Low	360 (35.8)
High	646 (64.2)
Online disinhibition, *n* (%)	
Low	477 (47.4)
High	529 (52.6)
Prior OGBV victimization, *n* (%)	
No	84 (8.3)
Yes	922 (91.7)

DASS-21 score categorization indicated anxiety is the most prevalent mental distress, observed in 41.5% of respondents to varying degrees of severity. Meanwhile, approximately 19.3% of respondents were categorized as depressed, and 9.1% were categorized as stressed in various degrees of severity. We also observe that more than half of respondents demonstrate high levels of intolerance to uncertainty (64.2%) and online disinhibition (52.6%).

Regarding OGBV (Table 2), we found nearly all (91.7%) respondents have had prior OGBV victimization at least once in their lives. Accordingly, we found a high level of OGBV perpetration with 61% of respondents admitting to having committed at least one type of OGBV once. Incessant messaging was the most reported form of OGBV committed (30.9%).

**Table 2:** Types and frequency of online gender-based violence perpetrated by respondents

Types of OGBV	Never	Once	Several times	Often	Very often
Sent messages to someone even after they told you to stop messaging them (weight = 1.5)	695 (69.1)	124 (12.3)	95 (9.4)	67 (6.7)	25 (2.5)
Sent threatening, insulting, or harassing messages to a “significant other” (weight = 2.0)	765 (76.0)	104 (10.3)	73 (7.3)	44 (4.4)	20 (2.0)
Sent threatening, insulting, or harassing messages to an acquaintance (weight = 2.0)	785 (78.0)	102 (10.1)	62 (6.2)	37 (3.7)	20 (2.0)
Sent threatening, insulting, or harassing messages to someone you do not/barely know (weight = 2.0)	787 (78.2)	96 (9.5)	68 (6.8)	34 (3.4)	21 (2.1)
Sent unwanted pornographic messages (weight = 2.0)	798 (79.3)	90 (8.9)	56 (5.6)	37 (3.7)	25 (2.5)
Called someone offensive names in a public online space (weight = 2.0)	783 (77.8)	104 (10.3)	69 (6.9)	34 (3.4)	16 (1.6)
Share private messages, videos, or images of a (non-romantic) friend publicly or with other friends (weight = 2.5)	701 (69.7)	139 (13.8)	107 (10.6)	41 (4.1)	18 (1.8)
Share private messages, videos, or images of an ex-partner publicly or with other friends (weight = 2.5)	769 (76.4)	111 (11.0)	72 (7.2)	41 (4.1)	13 (1.3)
“Doxxing” (i.e., post someone’s personal contact details online, e.g., home address, or phone number) (weight = 3.0)	764 (75.9)	106 (10.5)	76 (7.6)	40 (4.0)	20 (2.0)

Univariate analysis (Table 3) found that all independent variables were significantly associated with OGBV perpetration. On multivariate analysis (Table 3); however, only four factors were identified as independent risk factors of OGBV perpetration: age, gender, anxiety, online disinhibition, and prior OGBV victimization. Predictably, women and non-binary respondents were less likely to perpetrate OGBV with aOR OF 0.51 (95% CI 0.33–0.78). Older age was also associated with a lower likelihood of OGBV perpetration with an aOR of 0.95 (95% CI 0.92–0.98) per incremental year. Meanwhile, anxiety, online disinhibition, and OGBV victimization history were also significant risk factors for perpetration. Among those three, victimization was identified as the strongest risk factor with an aOR of 11.29 (95% CI 5.11–18.51).

**Table 3:** Bivariate and multivariate analysis for determinants of perpetrating OGBV

Variables (*n* = 1006)	Perpetrated OGBV		cOR (95% CI)	aOR (95% CI)
	No (***n*** = 392)	Yes (***n*** = 614)		
Age (years), median (IQR)	27 (23–31)	25 (21–29)	0.93 (0.91–0.96)**	0.95 (0.92–0.98)**
Gender, *n* (%)				
Men	40 (25.6)	116 (74.4)	1	1
Women and non-binary	352 (41.4)	498 (58.6)	0.49 (0.33–0.72)**	0.51 (0.33–0.78)**
Sexual orientation, *n* (%)				
Heterosexual	381 (39.9)	574 (60.1)	1	1
Non-heterosexual	11 (21.6)	40 (78.4)	2.41 (1.22–4.76)*	1.51 (0.71–3.18)
Depression category, *n* (%)				
No depression	344 (42.4)	468 (57.6)	1	1
Depressed	48 (24.7)	146 (75.3)	2.24 (1.57–3.19)**	1.18 (0.75–1.84)
Anxiety category, *n* (%)				
No anxiety	283 (48.0)	306 (52.0)	1	1
Anxiety	109 (26.1)	308 (73.9)	2.61 (1.99–3.43)**	1.82 (1.30–2.56)**
Stress category, *n* (%)				
No stress	372 (40.7)	542 (59.3)	1	1
Stressed	20 (21.7)	72 (78.3)	2.47 (1.48–4.13)**	1.23 (0.67–2.24)
Intolerance of uncertainty, *n* (%)				
Low	167 (46.4)	193 (53.6)	1	1
High	225 (34.8)	421 (65.2)	1.62 (1.25–2.11)**	0.90 (0.66–1.24)
Online disinhibition, *n* (%)				
Low	223 (46.8)	254 (53.2)	1	1
High	169 (31.9)	360 (68.1)	1.87 (1.45–2.42)**	1.38 (1.03–1.85)*
Prior OGBV victimization, *n* (%)				
No	72 (85.7)	12 (14.3)	1	1
Yes	320 (34.7)	602 (65.3)	11.29 (6.04–21.11)**	9.72 (5.11–18.51)**

*p < 0.05

**p < 0.01

Subgroup analysis was conducted including 614 respondents who admitted to perpetrating some type of OGBV at least once (Table 4). OGBV perpetration scoring found a median score of 7.50. The interquartile range was 3.00 to 19.50 although the overall range was from 1.50 to 78.00. It showed the distribution of the OGBV perpetration score was extremely skewed. Thus, the logistic transformation was conducted prior to linear regression analysis.

**Table 4:** Multivariate linear regression sub-group analysis for determinants of severity and frequency perpetrating OGBV among OGBV perpetrators

Covariates	Standardized coefficient (*β*)	*p*	Model summary		
			*R*	*R*^2^	*p*
Age	0.073	0.055	0.383	0.147	< 0.001**
Women and non-binary gender	− 0.207	< 0.001**			
Non-heterosexual orientation	− 0.005	0.891			
High depression	0.042	0.360			
High anxiety	0.169	< 0.001**			
High stress	0.048	0.265			
High intolerance of uncertainty	0.009	0.839			
High online disinhibition	0.115	0.005**			
Prior OGBV victimization	0.089	0.019*			

*p < 0.05

**p < 0.01

The result of multiple linear regression showed fewer independent determinants for frequency and severity of OGBV perpetration. Similar to prior logistic regression, women and non-binary respondents were associated with lower OGBV perpetration scores with a standardized coefficient of − 0.207. Among predictors of a high OGBV score, a history of victimization was found as the weakest one with a standardized coefficient of 0.089. Meanwhile, anxiety was found to be the strongest predictor of a higher OGBV score with a standardized coefficient of 0.169.

## Discussions

Our results indicated that a high proportion of Indonesian young adults have perpetrated some forms of OGBV at least once. We also identified several risk factors of OGBV perpetration which included younger age, male gender, anxiety, high online disinhibition, and prior history of OGBV victimization. Among those who have perpetrated OGBV, male gender, anxiety, online disinhibition, and prior victimization were associated with a higher OGBV perpetration score.

The risk factors of OGBV perpetration identified in this study indicated that it can be seen in a framework that combined perpetration of GBV, online harassment, and indeed violence in general. Anxiety especially has been identified as a general risk factor of violence and aggression (Chung et al. 2019). Social and biological mechanisms seemed to be behind those associations. From a biological standpoint, it has been postulated that control of anxiety and aggression may share overlapping neurological pathways in the brain (Neumann et al. 2010). This association is especially visible during adolescence, an age group associated with heightened aggressive behavior (Chung et al. 2019). This is supported by our data which showed a reduced likelihood of perpetration associated with older age.

Specific to GBV, aggression was associated with a specific concept of anxiety known as male or masculine anxiety (Eisler et al. 1988). It is a specific form of anxiety that arises due to a perceived threat to male dominance or the masculine gender role. Alternatively, it has also been defined as anxiety that emerges from a gap between the perceived self-masculinity and the perceived ideal masculinity. This definition of masculine anxiety has been described as early as 1988 (Eisler et al. 1988). More recently, the concept of male anxiety has been found in a qualitative study among male young adults in South Africa and male students of Asian descent in the USA (Wong et al. 2014; Colpitts 2019).

An interesting finding from our data is the fact that the respondents are predominantly women. As anxiety is found as an independent predictor of OGBV, it can be postulated that patriarchy-related anxiety may also explain this phenomenon. This has been previously described in a Chinese qualitative study (Tang 2021). Different from the masculine framework, however, patriarchy-related anxiety among women stems from a perceived threat from women who do not fulfill the patriarchal expectation. Internalized patriarchy led women to perceive non-conforming women to place themselves above other women, taking up the masculine role, which led to tension and anxiety (Opoku 2017).

In the context of the pandemic during which this survey took place, there are ample evidence that anxiety increased in all age groups during this period, including among young adults. Global meta-analyses estimated that the prevalence of anxiety disorders reached 25–29% during the pandemic, which may have tripled compared to the pre-pandemic period (Salari et al. 2020; Santabarbara et al. 2021). Indonesian studies reported similar figures with significant anxiety reported to reach around 20% of the population (Anindyajati et al. 2021). These results are similar to our findings of moderate to severe anxiety prevalence which reached around 25% of our respondents. At the same time, these reports of increased anxiety coincided with multiple reports of increased GBV incidence during the pandemic (Mittal and Singh 2020; Opanasenko et al. 2021). This indicated the pandemic situation might have exacerbated general anxiety in the population which may have contributed to GBV perpetration as well as OGBV.

At the same time, our findings showed that OGBV perpetration can also be predicted by known predictors of online violence. Online disinhibition has been a well-described predictor of online abuse and violence (Wachs and Wright 2018). This association has been described as early as 2004. It was enhanced by two characteristics of online interaction: anonymity and asynchronicity, which enable perceived dissociation from one’s online behavior (Suler 2004). Recently, the framework has been confirmed to predict various forms of online violence from cyberbullying to sexual harassment (Wachs and Wright 2018; Zhong et al. 2020).

Normalization of online violence also played a role in facilitating OGBV perpetration. Our data support this postulation from the findings that prior victimization is a risk factor for perpetration. Prior victimization may warp one’s perception of accepted normal behavior which manifests into acceptance of future victimization or perpetration (Debowska et al. 2021). Exposure to OGBV by prior victimization may also desensitize respondents. The link between desensitization through media exposure and perpetration has been established in prior studies (Krahé et al. 2015; Vossen et al. 2017).

These risk factors for online violence and cyberbullying might be exacerbated during the pandemic as more and more activities were conducted online. During the survey period, most workplaces and educational institutions have operated online for nearly 1 year. There have been studies that have shown that this arrangement facilitated an increased incidence of online violence in various countries (Jain et al. 2020; Yang 2020; Barlett et al. 2021). One study in particular attempted to address the role of online disinhibition in facilitating online violent behavior during the pandemic and found it has not increased (Barlett et al. 2021). Instead, it is inferred that increased online violence during the pandemic was more associated with attempts to cope with anxiety during the pandemic and sheer increased frequency of online activities (Jain et al. 2020; Barlett et al. 2021).

Our results identified risk factors of OGBV which can be traced to stem from predictors of GBV and online violence perpetration which has been exacerbated due to the pandemic situation. These results implied a need for a multistage, two-pronged, intervention program to improve the inadequacy of prior recommendations against OGBV that focused more on online moderation. Short-term efforts should be conducted to combat the exacerbation of OGBV perpetration during the pandemic. Improved content moderation currently makes up the main approach to this issue (Jhaver et al. 2018). While there has been improvement in content moderation, however, it can do little without support from actual law enforcement to deter OGBV perpetration with real-life consequences (Powell and Henry 2018).

Long-term approach should build around preventive efforts, especially building awareness of the issue and building a network of peers that would not normalize any form of online violence. Awareness-building problems should be devised in ways that decrease the risk of the perceived threat that comes from female empowerment. To minimize the perceived threat from such programs, we could co-opt a patriarchal hierarchical structure by engaging with traditional authority figures such as faith organizations (le Roux and Bowers-Du Toit 2017). We should also engage with men, at-risk groups for OGBV perpetration, by building supportive peer groups of gender-aware men (Greig et al. 2015).

Another interesting finding in our study is the high prevalence of OGBV perpetration despite predominantly female respondents. This implies the need to further investigate GBV perpetrated by women as a separate phenomenon that needs to be addressed. The current literature on GBV and OGBV is focused on men-perpetrated violence, which is the most prevalent type of GBV. However, the existing phenomena of GBV perpetrated by women may be severely underreported. Building a supportive peer for women is important for empowerment and building a gender-equal society (Tang 2021).

This study is not without its weaknesses. The high proportion of women among our respondents may skew our results since previous studies found that OGBV is more likely to be perpetrated by men. However, the significant inclusion of men in our respondents can hopefully alleviate this concern. Another weakness is the unavailability of socioeconomic data, such as education level and employment, which may affect violence perpetration. Regardless, to the authors’ knowledge, this study is one of the few to discuss the risk factor of OGBV perpetration as a separate behavioral phenomenon.

## Conclusions

We identified several risk factors of OGBV perpetration which include male gender, younger age, anxiety, online disinhibition, and prior victimization history. These risk factors can be explained by combining the framework of predictors for GBV and online violence perpetration, such as patriarchal anxiety and online dissociation which have been exacerbated during the COVID-19 pandemic. In the short term, it implied a need for improved content moderation and law enforcement against OGBV perpetration. In the long term, it implied the need to engage with at-risk groups for OGBV perpetration by co-opting their authority figures so as to not provoke further anxiety and aggression.

## Data Availability

The datasets used and/or analyzed during the current study are available from the corresponding author on reasonable request.

## Abbreviations

aOR: Adjusted odds ratio
cOR: Crude odds ratio
COVID-19: Coronavirus disease of 2019
DASS-21: Depression, Anxiety, and Stress Scale, 21 items version
GBV: Gender-based violence
IUS: Intolerance of Uncertainty Scale
ODS: Online Disinhibition Scale
OGBV: Online gender-based violence
OR: Odds ratio
